# Building the biomedical data science workforce

**DOI:** 10.1371/journal.pbio.2003082

**Published:** 2017-07-17

**Authors:** Michelle C. Dunn, Philip E. Bourne

**Affiliations:** Office of the Director, The National Institutes of Health, Bethesda, Maryland, United States of America

## Abstract

This article describes efforts at the National Institutes of Health (NIH) from 2013 to 2016 to train a national workforce in biomedical data science. We provide an analysis of the Big Data to Knowledge (BD2K) training program strengths and weaknesses with an eye toward future directions aimed at any funder and potential funding recipient worldwide. The focus is on extramurally funded programs that have a national or international impact rather than the training of NIH staff, which was addressed by the NIH’s internal Data Science Workforce Development Center. From its inception, the major goal of BD2K was to narrow the gap between needed and existing biomedical data science skills. As biomedical research increasingly relies on computational, mathematical, and statistical thinking, supporting the training and education of the workforce of tomorrow requires new emphases on analytical skills. From 2013 to 2016, BD2K jump-started training in this area for all levels, from graduate students to senior researchers.

## Background: Emergence of biomedical data science as a discipline

With biomedical research becoming increasingly data intensive, biomedical data science should likewise become increasingly prominent. Investigators are generating large, complex, multidimensional, and diverse datasets through new technologies developed in an age when sequencing a human genome costs under US$1,000, sensors are ubiquitous, and medical records are born digital. The era of big data has arrived in biomedical research.

Big data has brought with it new challenges, both technical and social. Challenges abound in sharing, accessing, and analyzing data as well as in drawing reliable conclusions from data. These challenges require research to develop a means of overcoming them, training to share the new knowledge with those who need to use it, and training of the next generation of data science researchers. Overcoming technical challenges to using biomedical data is the work of a biomedical data scientist.

The occupation called “data scientist” is a relatively recent invention, coined by D. J. Patil and Jeff Hammerbacher in 2008 [1] to describe a high-level professional who makes discoveries from copious amounts of messy data, using and building data products along the way. The tools of this occupation are quickly evolving, and with them, the realm of possibilities is expanding. As more examples of data science are produced, even more are imagined in new applications, making “data scientist” an occupation here to stay.

Professional training in data science, mostly at the master’s level, arose first under the name of “applied statistics,” then “analytics,” and now “data science.” Training in data science has emerged at all levels. At the undergraduate level, universities such as the University of Michigan and University of California, Berkeley launched data science majors in the fall of 2015. At the master’s level, North Carolina State University and the University of Virginia are just 2 examples of programs with professional master’s degrees that combine coursework and hands-on data experiences in a team environment. At the doctorate level, many institutions offer a PhD in related fields but with “concentrations” in data science. Although currently rare, leading-edge institutions such as University of Washington are even issuing PhDs called “Advanced Data Science” in a field.

Although it is undisputable that “data science” is a profession, whether it is an academic discipline is still under debate, not unlike the more mature discipline/profession called “bioinformatics.” Data science utilizes ideas from computer science, statistics, mathematics, engineering, physics, operations research, and library science and is deeply embedded in an application domain. One view is that data science is an umbrella term that covers a range of generalizable tools and techniques for extracting insight from data [2]. Another view, advocated by Berkeley professor Michael Jordan, is that data science is a unification of statistical and computational thinking, for example, proving conditions under which the quality of an inference improves with the data size, given a fixed amount of computation [3]. Stanford professor David Donoho argues that data science is the scientific study of “data analysis science wide”—that is, the field that studies how to improve learning from data [4]. While debate in public forums may eventually push these differing views to coalesce, what is clear now is that data science is in high demand and the talent supply is short [5].

Evidence for the high demand and low supply for data science can be found in the number of unfilled jobs and the high salaries. Recruiting firm BurtchWorks reports a median entry-level starting salary for data scientists at US$97,000 as of their April 2016 report [6]. Hiring platforms Glassdoor and Indeed both list over 100,000 open data science jobs in January 2017. Biomedical data science inherits the supply and demand gap from the more general category of data science. To close the supply and demand gap in the long run, training the next generation and retooling the current workforce are necessary but not sufficient. Closing the gap will also require retaining the workforce in biomedical data science—in a seller’s market, organizations with a culture that appreciates data science (e.g., by involving biomedical data scientists in leadership roles) will be in the best position to retain them.

Narrowing the gap between needed and existing biomedical data science skills has been a major focus of the National Institutes of Health (NIH) Big Data to Knowledge (BD2K) initiative. Education, training, and workforce development (“training,” for short) was 1 of the 5 recommendations of the Data and Informatics Working Group of the Advisory Committee to the NIH Director (http://acd.od.nih.gov/diwg.htm), which led to the launch of BD2K. As biomedical research increasingly relies on computational, mathematical, and statistical thinking, supporting the training and education of the workforce of tomorrow requires new emphases on analytical skills.

## Overview: BD2K efforts in biomedical data science

Although all NIH Institutes and Centers (ICs) share the goal and responsibility to support the biomedical workforce in acquiring necessary skills, the BD2K initiative acted for all ICs collectively to take an early step in the direction of data science. As such, the primary goals of training and education for the BD2K initiative are to

1. increase the number of biomedical data scientists by establishing biomedical data science as a career path;
2. expose biomedical scientists to data science
  - by supporting training opportunities, both in person and online,
  - by integrating data science into the curriculum,
  - by ensuring that training opportunities and resources are readily discovered and accessed;
3. increase diversity and outreach efforts;
4. foster collaborations between biomedical and data scientists.

To accomplish these goals, the BD2K initiative built up training efforts from 2013 through 2016 that provide a foundation for this new area of biomedical science. By creatively utilizing existing mechanisms to enable quick outreach to thousands of biomedical scientists, the BD2K initiative has laid the foundations to reach thousands more.

To get public input on which to base a diverse training and education portfolio, BD2K issued a Request for Information and held a workshop in June 2013 (summary). NIH then wrote a series of Funding Opportunity Announcements (FOAs), 16 in all (Table 1), to target diverse audiences. Through early 2017, the portfolio grew to about US$15 million,15% of the BD2K budget, and consisted of 71 grants and awards. In addition, 26 administrative supplements were issued in fiscal year 2016 to enhance the activities of existing grants and awards, totaling US$2.7 million. Collectively, the education and training opportunities reached an audience of varying experience levels, from novices to experts, and varying career levels, from undergraduates to senior faculty and instructors who will be teaching data science.

**Table 1:** A list of the 16 Funding Opportunity Announcements (FOAs), organized according to goal and the specific activity used to achieve the goal. In many cases, several approaches were used to make progress toward a goal, targeting subgroups by career level or science.

Goal	Activity	Title
		
Training Biomedical Data Scientists	Graduate Student Support	HG-14-004 Predoctoral Training in Biomedical Big Data Science (T32)
		HG-14-005 Revisions to Add Biomedical Big Data Training to Active Institutional Training Grants (T32)
		HG-14-006 Revisions to Add Biomedical Big Data Training to Active National Library of Medicine (NLM) Institutional Training Grants in Biomedical Informatics (T15)
		LM-16-002 The National Institutes of Health (NIH) Big Data to Knowledge (BD2K) Predoctoral Training in Biomedical Big Data Science (T32)
	Career Development for Faculty	HG-14-007 Mentored Career Development Award in Biomedical Big Data Science for Clinicians and Doctorally Prepared Scientists (K01)
		ES-16-002 NIH BD2K Mentored Career Development Award in Biomedical Big Data Science for Clinicians and Doctorally Prepared Scientists (K01)
		ES-16-003 NIH BD2K Mentored Career Development Award in Biomedical Big Data Science for Intramural Investigators (K22)
		
Exposing Biomedical Scientists to Data Science	Creating Open Educational Resource (OER) Content	HG-14-009 OERs for Biomedical Big Data (R25)
		HG-16-016 BD2K OERs for Skills Development in Biomedical Big Data Science (R25)
		LM-15-001 NIH BD2K Initiative Research Education: Massive Open Online Course (MOOC) on Data Management for Biomedical Big Data (R25)
		LM-15-002 NIH BD2K Initiative Research Education: OERs for Sharing, Annotating, and Curating Biomedical Big Data (R25)
	Holding In-Person Courses	HG-14-008 Courses for Skills Development in Biomedical Big Data Science (R25)
		ES-16-011 BD2K Research Education Curriculum Development: Data Science Overview for Biomedical Scientists (R25)
		
Diversity and Outreach	Undergraduate Research	MD-15-005 NIH BD2K Enhancing Diversity in Biomedical Data Science (R25)
		MD-16-002 NIH BD2K Enhancing Diversity in Biomedical Data Science (R25)
		
Foster Collaborations	Building Teams of Biomedical and Data Scientists	NSF-NIH Joint NSF/NIH Initiative on Quantitative Approaches to Biomedical Big Data (QuBBD)
		RFA-ES-15-004 NIH BD2K Biomedical Data Science Training Coordination Center (U24)
		
All	Building Teams; Resource Discovery	NIH BD2K Biomedical Data Science Training Coordination Center (U24)

## Training biomedical data scientists

Biomedical data scientists are expected to have deep expertise in data science and be conversant in the biomedical domain in which they are working. Early in this training, they are expected to go deep in data science and be biomedically “pluripotent”—obtaining basic biomedical knowledge in order to eventually specialize, e.g., in cancer biology. As the term is used here, data science consists of a wide range of expertise—therefore, depth in data science requires specialization and trade-offs. A freshly graduated biomedical data science PhD should not be expected to be as deep in the theory of statistics as a statistics PhD or as deep in algorithms as a computer science PhD. She will, however, have knowledge of a wide range of computational and statistical tools, techniques, and theory as well as exposure to biomedical science.

To increase the number of biomedical data scientists, new predoctoral training programs and new tracks in existing training programs have launched. BD2K currently funds 16 programs with up to 6 trainees each. What these programs require of students differs, reflecting the lack of agreement in the community about the discipline of data science. However, some knowledge and skills are common to them. While all programs require basic computing and statistical skills, most require whole courses in them and, in addition, a whole course in machine learning. About half the programs require second courses in advanced statistics and algorithms. Common statistical skills include inference and regression as well as exposure to the idea of controlling false discovery rates and to advanced modeling techniques such as using hidden Markov models. Common computational skills include programming, algorithms, optimization, high-performance computing, and data management.

Training certainly does not end at receiving a PhD in biomedical data science but also need not start with one. Biomedical data scientists may start with PhDs in other fields and then pick up the additional skills needed to do cutting-edge research in data science. Through mentored training, postdocs and junior faculty can start down a career path that holds much promise, supported by BD2K career development (K01 and K22) awards. The K01/K22 mechanisms were chosen in order to support the awardees for 3–5 years, which was felt to be the minimum amount of time needed to develop expertise. Because the program is new, long-term data on the success of the trainees is not yet available. However, these awardees have gathered a number of accolades, including receiving promotions and R01 awards. For example, Dr. Lana Garmire (Associate Professor, University of Hawaii) has become the principal investigator of 2 R01 grants and Dr. Jonathan Chen (Stanford) was promoted from a fellow to an Instructor of Medicine. These and other K01 and K22 awardees are well on their way to successful careers, whether in academia or industry.

Historically, the door separating academia from everywhere else has been viewed as a one-way door, but this antiquated view has to change. It is a hindrance to progress in new disciplines like data science, in which many of the problems and data driving the field come from the private sector or government. When skills are transportable between application areas, such as those in statistics, computer science, and engineering, knowledge gained while working on industry or public-sector applications is useful in academia. While salaries of industry may be attractive, the intellectual freedom to select challenges may draw some data scientists from industry to academia. Hence, biomedical data science should ensure that the door opens both ways. BD2K encourages the door to swing both ways by (1) ensuring that graduate trainees develop skills useful in industry and (2) enabling data scientists employed in industry to return to academia without a pay cut through making the salary cap on the BD2K K01 award much higher than other K01 awards (US$185,000/year as opposed to US$50,000–US$100,000). Movement in and out of academia could also be encouraged through data science research, i.e., the development and usage of productivity measures other than the number of academic publications. When data scientists move between academia, industry, and government, they cross fertilize in a way that helps all sectors—practical problems inspire new research, and new research is incorporated into practice.

## Training biomedical scientists to be conversant in data science

Not all biomedical scientists will become biomedical data scientists, nor should they. They should, however, have some familiarity with the ideas of data science, its power, and the complications and limitations that might arise. All biomedical scientists need to be conversant in data science; more broadly, leaders advocate that all college graduates need to be conversant in data analysis [7].

For established biomedical scientists to increase their data science knowledge, Massive Open Online Courses (MOOCs) and other Open Educational Resources (OERs) are flexible and readily accessible. Already, thousands of learners have benefited from them, and because they scale, thousands more easily could. Although only 13 OERs were supported by BD2K, many more data science OERs exist. However, finding them can be difficult due to their diverse forms and distributed locations. To help users find data science educational resources that are applicable to biomedical science, the Educational Resource Discovery Index (ERuDIte) is being built [8]. ERuDIte scrapes the web for educational resources, organizes them, and allows the user to create personalized learning pathways through biomedical data science based on free resources. ERuDIte promotes the usage of resources that are Findable, Accessible, Interoperable, and Reusable (FAIR) [9]. Many of the resources gathered by ERuDIte are crowdsourced from the immense online data science community built through the efforts of organizations such as Data and Software Carpentry and the Mozilla Foundation.

The BD2K initiative has a focus on open science, including open resources. As a guardian of public money, the NIH has a responsibility to use that money in a way that benefits as many people as possible. OERs, which are available to everyone, help to democratize information and allow all learners not only equal access to knowledge but also, paradoxically, personalized access. Learning no longer requires physically showing up at a particular time and place but instead can be done from the comfort of your home after putting the kids to bed. More efficient learning is within reach through personalized learning, in which content is automatically presented based on what you know and what you want to know, plugging holes in knowledge and strengthening weak spots.

While OERs and the personalized learning that they enable are particularly suited to established biomedical researchers, biomedical PhD students should gain exposure to data science through university coursework. Because universities develop and maintain training programs, they know best what trade-offs must be made to add new experiences to that training. To enable them to easily add data science awareness into the repertoire of biomedical trainees, BD2K issued an FOA [10] to encourage universities to work collaboratively on curriculum development. Because this is a new area of science in which there is not yet agreement on the necessary knowledge and skills, a collaborative approach would allow institutions to learn from one another while still enabling them to maintain their independence.

Being conversant in data science may start in graduate school but could continue through OERs, short courses, or other on-the-job training, if necessary. Data science fluency is critical because many biomedical problems will be data intensive and hence require teams that include data scientists.

## Building biomedical data science teams

Because making biomedical advances requires understanding and controlling complex systems, no one has a complete understanding of every detail of a problem or its solution space. When problems or their solutions involve data, the future of biomedical science is in diverse teams of scientists whose expertise falls on the spectrum from being conversant in data science to having deep expertise. Training biomedical scientists to be conversant in data science and training biomedical data scientists provides the raw material for these teams. However, this is not enough. Even if a team has a common goal and complementary personalities, much effort is required to forge a team out of people from very different fields. For example, the team members must learn to speak a common language, learn what motivates each other, and understand what each side brings to the table.

To directly encourage the development of teams of biomedical scientists and data scientists, the BD2K initiative sponsored a couple of experiments, a series of innovation labs [11], and the RoadTrip program [12].

Data science innovation labs aim to catalyze teams of junior biomedical and data scientists. Innovation labs are mentored and facilitated week-long workshops that are designed to spark creativity and catalyze new teams. BD2K-sponsored innovation labs focused on bringing data scientists together with biomedical scientists to work on precision medicine (2015), mobile health (2016), and microbiome (2017) problems. Sponsored jointly by the National Science Foundation (NSF) Division of Mathematical Sciences and the NIH, these labs were facilitated by KnowInnovation and organized by a community-focused organization—the Statistical and Applied Mathematical Sciences Institute [13] in 2015 and the BD2K Training Coordination Center [14] (TCC) in 2016 and 2017. Over the course of the week, the approximately 30 participants (split evenly between mathematics, statistics, and biomedical science) worked first collectively and later as small teams to identify the problem space, brainstorm solutions, and develop research agendas. Facilitators ensured that this process happened efficiently by asking the pointed questions that drew participants past thinking about the ordinary and all the way to the creative. Mentors who are senior experts in either data science or the biomedical focus area gave feedback on the teams’ research agendas. Instead of having to go through a 6-month grant peer review process to get the feedback, the teams presented their work and got immediate feedback, enabling them to iterate quickly. The small interdisciplinary teams that formed were encouraged to apply for NSF or NIH funding, e.g., through the joint NSF-NIH Quantitative Biomedical Big Data program. Many of these teams successfully competed for funding, with success rates being much higher for lab participants than the general pool in the first year. Impartial evaluation by a review panel coupled with positive feedback from participants testifies to the success of the innovation lab.

Innovation labs are designed to bring together junior investigators from the biomedical and data sciences. To reach more senior data scientists, other tactics are needed. The RoadTrip program from the BD2K TCC is an example of a lightweight effort to catalyze the development of teams of data scientists and junior biomedical scientists. The goal of the RoadTrip program is to match junior biomedical scientists with senior data scientists for potential collaborations around the research problems of the biomedical scientist. Small awards are being made to offset travel costs associated with exploring these collaborations.

The innovation labs and the RoadTrip program are 2 of the many possible experiments to catalyze new teams of biomedical and data scientists and to help the team members overcome the initial barriers of working together.

## Analysis and future directions

Anticipating the skills that the workforce of tomorrow needs is fraught with uncertainty. Tools change and new knowledge is discovered, making best practices of yesterday outdated. Building a biomedical research workforce today to meet its needs tomorrow requires skating to where the puck will be, with inertia pulling in the opposite direction. The inertia is not necessarily due to lack of foresight or will but to competing demands for time and money. For this reason, the NIH has a responsibility to promote forward-thinking workforce development, as it has done in the BD2K initiative. NIH is not alone in this endeavor. Other funding agencies and foundations have their own approaches, which for funding agencies like NIH are tied to their existing funding mechanisms. On the other hand, foundations have more flexibility. For example, the Gordon and Betty Moore Foundation and the Alfred P. Sloan Foundation worked early and together to create data science environments at 3 institutions [15], which emphasized a new interdisciplinary way of thinking, with data science at its core. Likewise, the NSF established the Research Traineeship Program [16] seeking to train but also to provide innovative approaches to training. Support for training of data scientists across the national and international funding spectrum will only increase to meet demand.

The BD2K initiative had a strong emphasis from the beginning on training, aiming to dedicate 20% of its budget to it. No NIH IC, save the National Library of Medicine (NLM), spends even close to that percentage of its extramural budget on training. In the end, only about 15% of the BD2K budget was spent on training due to the lack of fundable applications.

On the surface, the lower-than-desired number of fundable applications was due to not having enough applications. But why were there so few applications? There could be several reasons:

1. There are not enough data scientists for the demand—for service work, perhaps the existing community prioritizes doing routine analyses over teaching.
2. The community doesn’t know how to do or review data science training because the science is not mature enough.
3. The award budget and duration was not big or long enough for the amount of work involved in bringing together interdisciplinary teams to develop and teach courses.
4. Advertisement of FOAs was inadequate.

Likely, the answer is a combination of all these reasons and perhaps more. Some of these will resolve themselves with time as coalescence around biomedical data science as a discipline happens and the word that NIH is interested in biomedical data science spreads. The limitation about the size and duration of awards, while a constraint of using the request for applications, can be modified in any future funding calls. Balancing the disappointment of not funding more awards is the knowledge that the ones funded are of very high quality and are producing resources and training people that will enable good science for years to come.

The BD2K initiative has accomplished a great deal in 4 years, but continued progress requires leadership and a sustained commitment of resources, both staff and grant funding. Each of the areas described above needs more work.

Training biomedical scientists to be conversant in data science relies on the integration of basic data science literacy into required coursework and opportunities for continued training throughout a lifetime, e.g., through OERs and personalized learning. Training biomedical data scientists through graduate school and postdoctoral studies is needed, but they need places to land—jobs that utilize their skills and allow growth. Developing suitable and desirable career paths for biomedical data scientists requires change at universities; it requires universities to value their contributions to an important societal problem rather than solely valuing contributions to the core of the field. Some institutions like Carnegie Mellon University and the University of Virginia have long nurtured an interdisciplinary culture and are capitalizing on that culture to excel in the field of data science.

Many of the programs put into place require leadership to reap full benefits toward building the biomedical workforce of the future. That leadership needs to come from all areas, whether at the NIH or other government agencies, at universities or research institutes, or in industry. Data science is here to stay—data are becoming more ubiquitous, not less, and reaping the benefits of that data requires good science.

## Abbreviations

BD2K: Big Data to Knowledge
ERuDIte: Educational Resource Discovery Index
FAIR: Findable, Accessible, Interoperable, and Reusable
FOA: Funding Opportunity Announcement
IC: Institute and Center
MOOC: Massive Open Online Course
NIH: National Institutes of Health
NLM: National Library of Medicine
NSF: National Science Foundation
OER: Open Educational Resource
QuBBD: Quantitative Approaches to Biomedical Big Data
TCC: Training Coordination Center
